# Signal transducer and activator of transcription-3 drives the high-fat diet-associated prostate cancer growth

**DOI:** 10.1038/s41419-019-1842-4

**Published:** 2019-09-02

**Authors:** Hiu Yee Kwan, Bin Liu, Chunhua Huang, Sarwat Fatima, Tao Su, Xiaoshan Zhao, Alan H. M. Ho, Quanbin Han, Xianjing Hu, Rui-Hong Gong, Minting Chen, Hoi Leong Xavier Wong, Zhaoxiang Bian

**Affiliations:** 1 Centre for Cancer and Inflammation Research, School of Chinese Medicine, Hong Kong Baptist University, Hong Kong, China; 2 Department of Cardiology, The Second Affiliated Hospital of Guangzhou Medical University, Guangzhou, Guangdong 510260 China; 3 International Institute for Translational Chinese Medicine, Guangzhou University of Chinese Medicine, Guangzhou, Guangdong 510006 China; 4 School of Traditional Chinese Medicine, Southern Medical University, Guangzhou, Guangdong 510260 China

**Keywords:** Cancer microenvironment, Prostate cancer

## Abstract

Prostate cancer (PCa) is the second leading cause of cancer death in men. PCa progression can be associated with obesity. Signal transducer and activator of transcription-3 (STAT3) plays a crucial role in PCa growth. However, whether STAT3 plays a role in high-fat diet (HFD)-associated PCa growth is unknown. Our data show that HFD feeding increases tumor size, STAT3 phosphorylation, and palmitic acid (PA) level in the xenograft tissues of the PCa-bearing xenograft mouse model. In vitro studies show that PA increases STAT3 expression and phosphorylation (STAT3-Y705) in PCa. Computational modeling suggests strong and stable binding between PA and unphosphorylated STAT3 at R593 and N538. The binding changes STAT3 structure and activity. Functional studies show that both STAT3 mutants (R583A and N538A) and STAT3 dominant negative significantly reduce PA-enhanced STAT3 phosphorylation, PA-increased PCa cell proliferation, migration, and invasion. In the xenograft mouse models, the HFD-increased tumor growth and STAT3 phosphorylation in tumors are reversed by STAT3 inhibition. Our study not only demonstrates the regulatory role of PA/STAT3 axis in HFD-associated PCa growth but also suggests a novel mechanism of how STAT3 is activated by PA. Our data suggest STAT3 as a therapeutic target for the treatment of HFD-associated PCa.

## Background

Prostate cancer (PCa) is the most common cancer and the second leading cause of cancer death among men^1^. The National Cancer Institute reported that ~11.2% of men will be diagnosed with PCa at some point during their lifetime^2^. Androgen deprivation treatment reduces symptoms in about 70–80% of patients with advanced PCa, but most tumors relapse within 2 years to an incurable androgen-independent state. Furthermore, PCa progression has been reported to be associated with obesity. A meta-analysis study encompassing 3,569,926 PCa patients showed that obesity is significantly correlated with the aggressive PCa and increased risk of the PCa-associated mortality^3^.

High-fat diet (HFD) affects PCa growth by changing the tumor microenvironment. HFD increases the myeloid-derived suppressor cells fraction and the M1/M2 macrophage ratio in the tumor, and the tumor growth is associated with interleukin-6 (IL-6) secreted by the prostatic macrophages^4^. Fatty acids in the HFD, especially palmitic acid (PA), affect the LNCaP xenograft tumor growth by elevating the expression of macrophage inhibitory cytokine^5^.

PCa cells can uptake the fatty acids from extracellular space as demonstrated by an experiment with Fourier transform infrared micro-spectroscopy in which the PCa cells uptake isotopically labeled fatty acids from adipocytes^6^. Interestingly, PCa cells have a dominant uptake of fatty acids over glucose for energy production^7^. This re-programmed metabolism is unique to PCa, and further implicates the role of HFD or dietary fat per se on PCa growth.

Signal transducer and activator of transcription-3 (STAT3) is a transcription factor; its aberrant activation is observed in ~50% of PCa patients^8^. STAT3 can be activated by cytokines and growth factors. For example, in the patients, the elevated high circulating levels of IL-6 activate STAT3^9^. STAT3 plays a crucial role in PCa cell proliferation, survival, and differentiation^10,11^. STAT3 also facilitates immune evasion of PCa by negatively regulating cellular and innate immune response^12–15^. Nevertheless, whether STAT3 plays in a role in HFD-associated PCa growth has never been explored.

## Results

### STAT3 phosphorylation is increased in the xenograft tissues of the PCa-bearing xenograft mouse model under high-fat dietary intervention

**Fig. 1:**
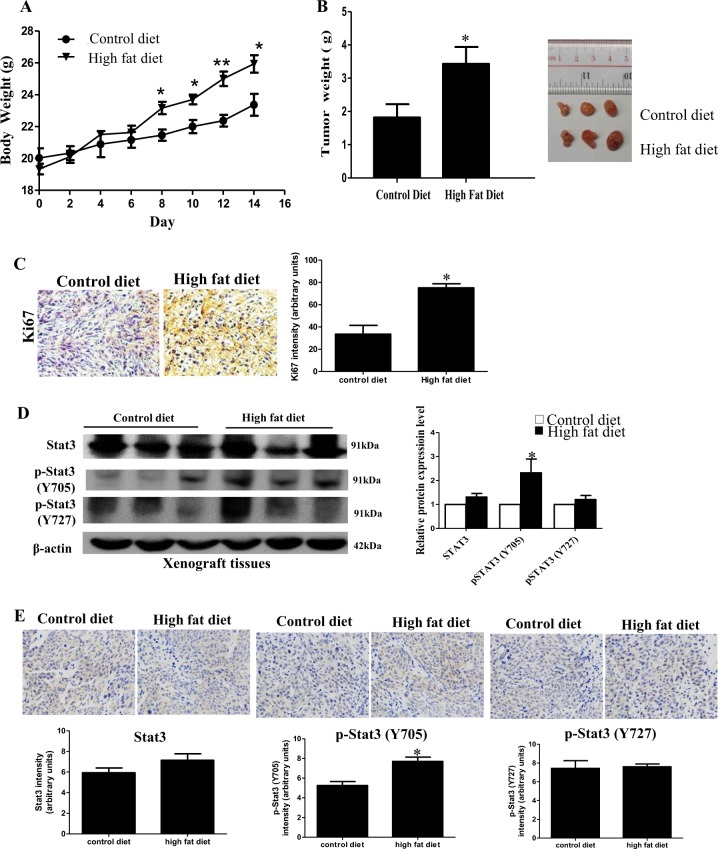
Signal transducer and activator of transcription-3 (STAT3) phosphorylation is increased in the xenograft tissues of the prostate cancer (PCa)-bearing xenograft mouse model after high-fat diet (HFD) feeding. **a** Body weight and **b** tumor weight of the DU145-bearing xenograft mouse models after feeding the HFD or matched control diet (CD) for 14 days. **c** Expression of Ki67 in the xenograft tissues. **d** Western blots and **e** immunohistochemistry staining of Stat3 and phosphorylated Stat3 (p-Stat3) at Y705 or Y727 in the xenograft tissues of the HFD-feeding and CD-feeding xenograft mouse models. Shown is the mean ± SEM, 4–10 mice in each group. *P value < 0.05

We first established a HFD-associated PCa xenograft mouse model to study the role of STAT3 in HFD-associated PCa growth. We subcutaneously inoculated DU145 cells in the nude mice, which were then randomly divided into HFD-feeding group or a matched control diet (CD)-feeding group. After 2 weeks of dietary intervention, the body weight (Fig. 1a) and the tumor weight (Fig. 1b) of the HFD-feeding mice were significantly greater than those of the CD-feeding mice. The expression of proliferation marker Ki67 in the xenograft tissues was also higher in the HFD-feeding mice than those in the CD-feeding mice (Fig. 1c), suggesting HFD increases PCa growth in our xenograft mouse model. Interestingly, phosphorylation of STAT3 at Y705 (p-STAT3-Y705) in the xenograft tissues was increased in the HFD-feeding group compared to CD-feeding group, while phosphorylation at Y727 was not significantly affected (Fig. 1d, e).

### HFD feeding increases PA level in the xenograft tissues of the PCa-bearing xenograft mouse model, and PA increases STAT3 phosphorylation (p-STAT3-Y705) in PCa

Next, we examined how HFD feeding increased STAT3 phosphorylation. Since the fatty acid contents are different between HFD and CD (Supplementary Table S1), we first explored whether the fatty acid contents in the xenograft tissues were affected by the dietary interventions, and whether the changes in these fatty acid levels affected STAT3 phosphorylation.

**Fig. 2:**
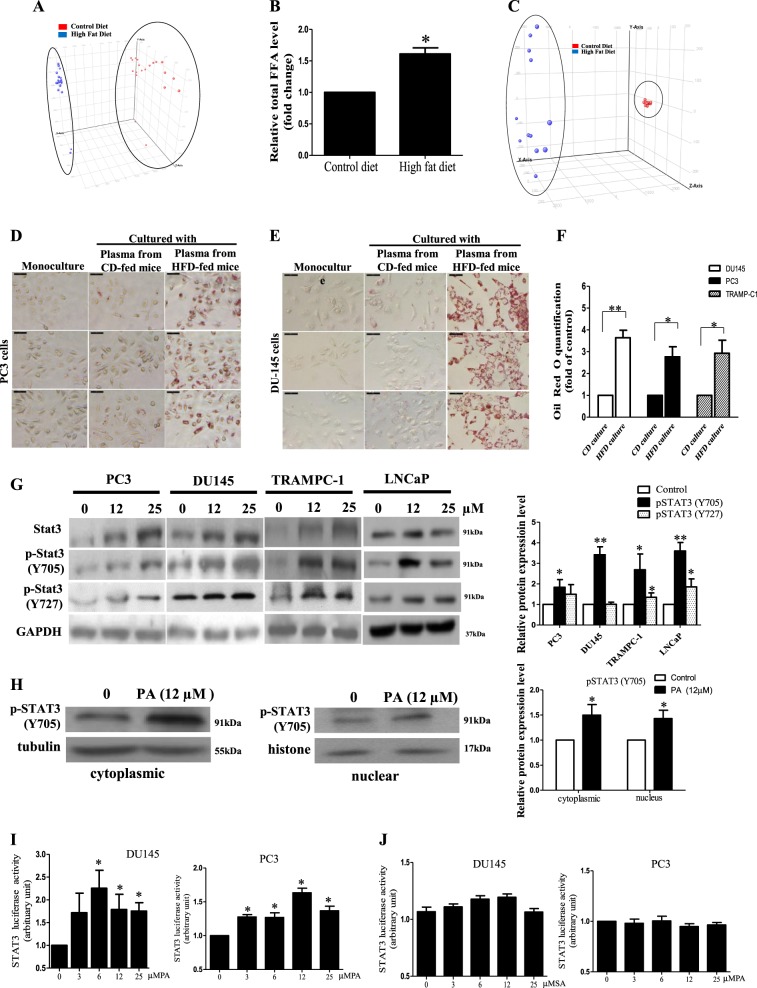
High-fat diet (FD) feeding increases palmitic acid (PA) level in the xenograft tissues of the prostate cancer (PCa)-bearing xenograft mouse model and PA increases signal transducer and activator of transcription-3 (STAT3) phosphorylation (p-STAT3-Y705) in PCa. **a** Principal component analysis and **b** relative total free fatty acid levels of the xenograft tissues of the mice. Shown is the mean ± SEM, 10 mice in each group. *P value <0.05. **c** Principal component analysis of the lipids in the serum of the DU145-bearing xenograft mouse models fed by HFD or control diet (CD), 5–6 mice in each group. Oil red-O staining of the lipids in **d** PC3 and **e** DU145 cells after culturing with the serum of the HFD-feeding or CD-feeding mice. **f** Oil red-O quantification of these cancer cells. CD culture, cells cultured with serum of CD-feeding mice; HFD culture, cells cultured with serum of HFD-feeding mice. **g** Western blots showing the levels of Stat3 and pStat3 at Y705 and Y727 in PCa cells upon PA challenge for 24 h. **h** Western blots showing the levels of p-Stat3 (Y705) in the cytoplasm and the nucleus of DU145 cells upon PA challenge. **i** Stat3-luciferase reporter activity upon PA challenge and **j** upon stearic acid challenge in DU145 and PC3 cells, respectively. Shown is the mean ± SEM, *n* = 3 independent experiments. *P value < 0.05 and **p value < 0.01

We used lipidomics to examine the lipid profiles in the xenograft tissues. Entities with high scores for particular component followed the expression pattern are shown in the principal component analysis (PCA). We found that the lipid metabolites of the xenograft tissues (Fig. 2a) showed distinct clustering between the HFD group and the CD group, suggesting that the HFD-feeding mice have a different xenograft lipidomic profile from the CD-feeding mice. The lipid species in the xenograft tissues that were modulated by the HFD feeding were listed in Supplementary Table S2. We also found that HFD feeding increased the total fatty acid levels in the xenograft tissues (Fig. 2b), which included PA (Table 1A). Besides, the dietary intervention also affected the serum lipid profile of the mice. The lipid metabolites of the serum showed distinct clustering between the HFD group and the CD group (Fig. 2c), suggesting the HFD-feeding mice have a different serum lipidomic profile from the CD-feeding mice. In particular, the levels of saturated fatty acids such as PA and stearic acid were significantly higher in the serum of HFD-feeding mice than those in the CD-feeding mice (Table 1B).

**Table 1A:** Concentrations of PA in xenograft tissues of the HFD-feeding and CD-feeding mice

Compound		Retention time (min)	µg/g tissue		Accurate MS
			CD	HFD	
Palmitic acid (16:0)	C_16_H_32_O_2_	17.7303	3.6180 ± 0.113	5.1820 ± 0.328*	256.2426

Shown is the mean plus or minus SEM, 10 mice in each group

*HFD* high-fat diet, *CD* control diet, *PA* palmitic acid, *MS* Mass Scientific

*P value < 0.05 between HFD-feeding group and CD-feeding group

**Table 1B:** Concentrations of PA and stearic acid in the serum of the HFD-feeding and CD-feeding mice

Compound		Retention time (min)	µg/ml		Accurate MS
			CD	HFD	
Palmitic acid	C_16_H_32_O_2_	17.7303	3.5710 ± 0.1346	7.4004 ± 0.272**	256.2426
Stearic acid	C18H36O2	21.4350	2.9420 ± 0.6544	3.5477 ± 0.678*	284.2707

Shown is the mean plus or minus SEM, 10 mice in each group

*HFD* high-fat diet, *CD* control diet, *PA* palmitic acid, *MS* Mass Scientific

*P value < 0.05, **p < 0.01 between HFD-feeding group and CD-feeding group

Next, we used ex vivo model to study whether PCa cells would uptake exogenous fatty acids from serum. In the ex vivo experiment, we cultured the PCa cells with the mouse serum^5,16^. We found that the PCa cells cultured with HFD-feeding mouse serum had significantly higher lipid contents than those cultured with CD-feeding mouse serum or those cultured without serum (Fig. 2d, f). Interestingly, DU145, PC3, and TRAMP-C1 cells cultured with HFD-feeding mouse serum had elevated levels of PA (Table 2A). However, although stearic acid level in HFD-feeding mouse serum was elevated, only DU145 cells cultured with the serum had a significant increase in stearic acid level (Table 2B), but not the PC3 and TRAMP-C1 cells (Table 2B).

**Table 2:** Quantification of PA and stearic acid levels in PCa cells after culturing with serum

Compound	Retention time (min)	Accurate MS	PCa cells	ng/mg protein	
				Monoculture	Co-culture with serum from HFD-fed mice
A					
Palmitic acid	17.7303	256.2426	TRAMP-C1	10.764 ± 0.325	12.081 ± 0.481*
C_16_H_32_O_2_			DU145	7.580 ± 0.308	10.847 ± 0.183*
			PC3	9.891 ± 0.273	10.939 ± 0.202*
B					
Stearic acid	21.4350	284.2707	TRAMP-C1	6.091 ± 0.535	7.618 ± 1.056
C_18_H_36_O_2_			DU145	25.321 ± 1.73	39.382 ± 0.609*
			PC3	6.375 ± 0.0.618	5.832 ± 0.432

Concentrations of (A) PA and (B) stearic acid in the DU145, PC3, and TRAMP-C1 cells after culturing with or without the serum of the HFD-feeding mice. Shown is the mean plus or minus SEM, *n* = 3 independent experiments

*HFD* high-fat diet, PCa prostate cancer, *PA* palmitic acid, *MS* Mass Scientific

*P value < 0.05 between control and serum-cultured groups

We then explored whether PA affected STAT3 expression and activity in the PCa. Data showed that PA significantly increased STAT3 protein expression in mouse PCa cells and the human castration-resistant PCa cells such as PC3 and DU145 cells, but not in the androgen-sensitive PCa cells such as LNCaP cells (Fig. 2g). Nevertheless, PA significantly increased STAT3 phosphorylation at Y705 in all the tested PCa cell lines (Fig. 2g). However, p-STAT3-Y727 was only significantly increased in PC3 and LNCaP cells upon PA challenge (Fig. 2g). These data suggest that PA increases STAT3 activity. Indeed, the nuclear localization of p-STAT3-Y705 was also increased in the PCa cells upon PA challenge (Fig. 2h). Reporter activity assay with STAT3 reporter construct 4xM67 pTATA TK-Luc (Addgene) showed that PA (Fig. 2i), but not stearic acid (Fig. 2j), increased STAT3 activity. Taken together, these data suggest that PCa cells take up exogenous PA, which increases STAT3 phosphorylation and activity in the PCa cells.

### PA increases STAT3 transcription

**Fig. 3:**
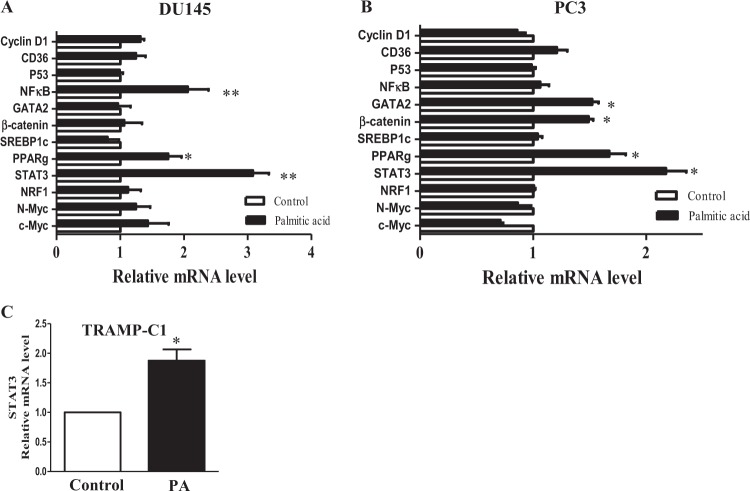
Palmitic acid (PA) increases signal transducer and activator of transcription-3 (STAT3) transcription. Relative messenger RNA (mRNA) levels of the oncogenic genes in **a** DU145 and in **b** PC3 cells upon PA challenge (12 µM) for 24 h. Cyclin D1, cluster of differentiation 36 (CD36), p53, nuclear factor-κB (NF-κB), GATA-binding protein 2 (GATA2), β-catenin, sterol regulatory element-binding protein-1 (SREBP-1), peroxisome proliferator-activated receptor-γ (PPAR-γ), Stat3, nuclear respiratory factor-1 (NRF-1), N-myc, and c-Myc. **b** The relative Stat3 mRNA levels in TRAMP-C1 cells upon PA challenge. Shown is the mean ± SEM, *n* = 3 independent experiments. *P value < 0.05 and **p value < 0.01

To explore the possible mechanism of action underlying how PA increased STAT3 protein expression in PC3 and DU145 cells, we first examined whether PA increased STAT3 gene transcription. Since it has been reported that PA increases nuclear factor-κ light chain enhancer of activated B cells (NF-κB) expression^17^, we used NF-κB as a positive control in this study. PCR array data suggest that PA significantly increased the messenger RNA (mRNA) levels of STAT3 and peroxisome proliferator-activated receptor-γ (PPAR-γ) in both DU145 (Fig. 3a) and PC3 cells (Fig. 3b). The upregulation of STAT3 mRNA after PA treatment was also observed in TRAMP-C1 cells (Fig. 3c). However, PA did not affect the expressions of other oncogenes in these cells, such as NF-κB, cyclin D1, CD36, TP53, GATA-binding protein 2 (GATA2), β-catenin, sterol regulatory element-binding protein 1, nuclear respiratory factor-1 (NRF-1), N-myc, and c-Myc (Fig. 3a, b). These data suggest that the increased STAT3 protein level upon PA challenge in PCa cells may due to the increased STAT3 mRNA level.

### PA has strong and stable binding with unphosphorylated STAT3, which changes the protein structure of the unphosphorylated STAT3

Our data suggest that PA increases STAT3 protein level, which subsequently increases the amount of phosphorylated STAT3 in PC3, DU145, and TRAMPC-1 cells. However, in LNCaP cells, PA increases the STAT3 phosphorylation without increasing STAT3 total protein level. These interesting results imply that there are parallel mechanisms of action underlying the enhanced STAT3 phosphorylation in PCa cells upon PA challenge.

Since STAT3 phosphorylation can be mediated by Janus kinase (JAK2)^10^ or other upstream signaling molecules such as epidermal growth factor receptor (EGFR) and extracellular signal-regulated kinase (ERK)^11,18^, we first investigated whether JAK, EGRF, and ERK activities mediated the PA-increased STAT3 phosphorylation in PCa. We found that PA treatment did not affect JAK2 phosphorylation at Y1007/1008 residues (Supplementary Fig. S1A), EGFR phosphorylation at Y845/Y992/Y1068/Y1148 residues (Supplementary Fig. S1B), and ERK phosphorylation (Supplementary Fig. S1C), suggesting that the enhanced STAT3 phosphorylation in PCa is not due to enhanced JAK, EGFR, and ERK activities. Besides, we also examined the level of IL-6^19^ and the activity of leptin receptor^20^, which are known to activate STAT3. However, PA treatment did not significantly affect the level of IL-6 (Supplementary Fig. S1D) or phosphorylation of leptin receptor (Supplementary Fig. S1E).

**Fig. 4:**
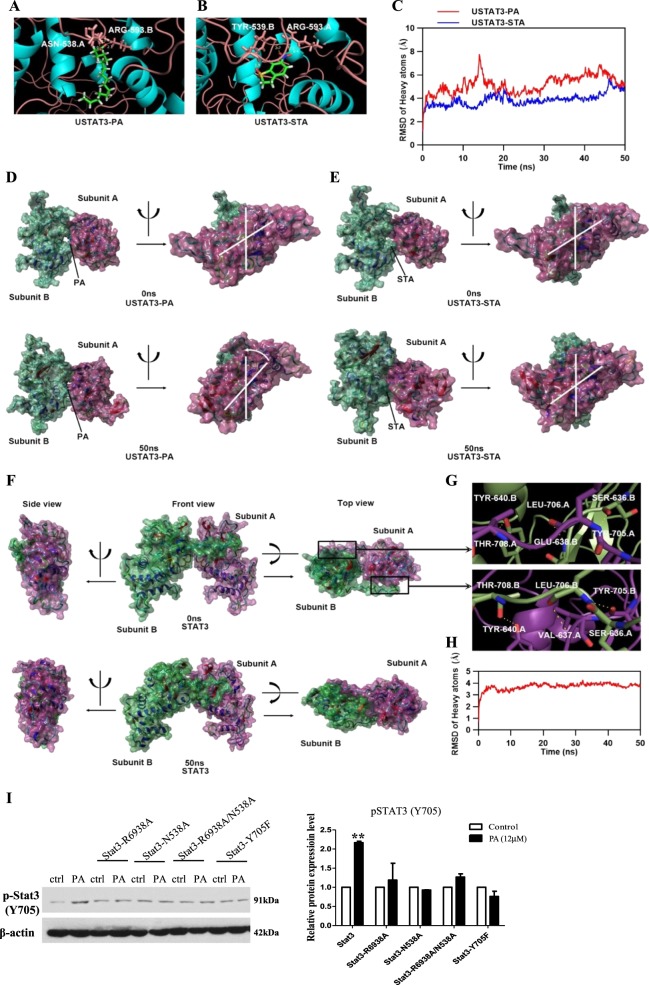
Palmitic acid (PA) has strong and stable binding with unphosphorylated signal transducer and activator of transcription-3 (USTAT3), which changes the protein structure of the USTAT3. **a–e** Molecular simulations showing the interaction of USTAT3 with PA or stattic (STA), a specific Stat3 inhibitor. **a** Three-dimensional ribbon model of PA in complex with USTAT3. **b** Three-dimensional ribbon model of STA in complex with USTAT3. **c** Plots of root mean square deviation (RMSD) of heavy atoms of USTAT3-PA (red) and USTAT3-STA complex (blue). **d** Surface presentation of the USTAT3-PA complex crystal structure at 0 and 50 ns. **e** Surface presentation of the USTAT3-STA complex crystal structure at 0 and 50 ns. **f** Surface presentation of the homology model of human STAT3 dimer at 0 and 50 ns. **g** Binding mode of subunit A’s transactivation domain with subunit B’s SH2 domains (up) and subunit A’s SH2 domains with subunit B’s transactivation domain (down). **h** Plots of RMSD of heavy atoms of human STAT3 dimer. **i** Western blot showing the expressions of phosphorylated (p)-Stat3 (Y705) in DU145 cells, or DU145 cells overexpressed with Stat3-R6938A, Stat3-N538A, Stat3-R6938A/N538A, or Stat3-Y705F after treating with PA for 24 h

To explore other possible mechanisms of action underlying how PA increased STAT3 phosphorylation in PCa cells, we examined whether there was a direct interaction between PA and STAT3, which may facilitate STAT3 phosphorylation and activation. To investigate the interaction between PA and unphosphorylated STAT3 (USTAT3), we performed molecular docking and molecular dynamics (MD) by using ICM-Pro and YASARA. The molecular docking between USTAT3 and STA, a STAT3 inhibitor, was also performed as control. The binding energy of USTAT3-PA and USTAT3-STA complex were −24.98 and −18.57 kcal/mol, respectively. The three-dimensional ribbon model of the USTAT3-PA and USTAT3-STA complex were presented in Fig. 4a, b, respectively. Hydrogen bond was formed between ASN-538 of USTAT3 subunit A and PA, and the distance of this hydrogen bond was 2.0 Å. Two hydrogen bonds were formed between ARG-593 of USTAT3 subunit B and PA, and the distance of these hydrogen bonds were 1.9 and 2.1 Å, respectively. Two hydrogen bonds were formed between TYR-539 of USTAT3 subunit B and STA, and the distance of these hydrogen bonds were 1.9 and 2.7 Å, respectively. Another two hydrogen bonds were formed between ARG-593 of USTAT3 subunit A and STA, and the distance of these hydrogen bonds were 2.3 and 2.4 Å, respectively. MD simulation results were presented in Fig. 4c–e. The root mean square deviation (RMSD) of USTAT3 proteins bound with PA was different from that of USTAT3-STA complex during 50 ns MD simulation (Fig. 4c). The surface presentation of the USTAT3-PA and USTAT3-STA complexes at 0 and 50 ns were presented in Fig. 4d, e. We found that PA and STA steadily presented at the binding site between USTAT3 subunits until the end of MD simulation. The relative position of two USTAT3 subunits was changed when binding with PA (Fig. 4d right), while the relative position of two USTAT3 subunits was stable when binding with STA (Fig. 4e, right). These molecular simulation results suggest that PA directly binds to USTAT3 protein and changes the USTAT3 conformation.

To investigate whether the changes of USTAT3 structure might facilitate the activation of STAT3, we generated a computational homology model of human STAT3 dimer. The surface presentation of the STAT3 homology model was presented in Fig. 4f. We found STAT3 subunit A and B were in the same horizontal plane from the side and top view. Interestingly, we found that transactivation domain of subunit A or B interacted with each others SH2 domain. Three hydrogen bonds were formed between TYR-640, GLU-638, and SER-636 of the subunit B’s transactivation domain and THR-708, LEU-706, and TYR-705 of the subunit A’s SH2 domain (Fig. 4g, up). The distance of these hydrogen bonds was 3.5, 3.4, and 3.2 Å, respectively. Accordingly, three hydrogen bonds were also formed between TYR-640, GLU-637, and SER-636 of the subunit A’s transactivation domain and THR-708, LEU-706, and TYR-705 of the subunit B’s SH2 domain (Fig. 4g, down). The distance of these hydrogen bonds were 3.5, 3.3, and 3.6 Å, respectively. MD simulation result of STAT3 homology model shows that the relative position of subunits A and B were stable during 50 ns MD simulation. The RMSD of human STAT3 dimer was raised in first 5 ns and fluctuated around 3.5 Å during last 45 ns MD simulation (Fig. 4h). These results indicated that subunits A and B of STAT3 kept in a same horizontal plane might be a key character of activated STAT3.

Since several potential binding sites on STAT3 are suggested for the binding with PA in our computational modeling, we constructed single-point mutation at each of these potential binding site, they are R593A and N538A. We also constructed double-point mutations R593A + N538A. We separately overexpressed these mutant constructs in PCa cells. As a negative control, we overexpressed the dominant-negative STAT3-Y705F^21^ in PCa cells. We found that PA could not increase the phosphorylation of these STAT3 mutants and STAT3 dominant negative (Fig. 4i). These results suggest that the potential binding sites R593 and N538 on STAT3 are important for the activation of STAT3 upon PA challenge.

### PA increases PCa cell proliferation, which is mediated by STAT3 activity

**Fig. 5:**
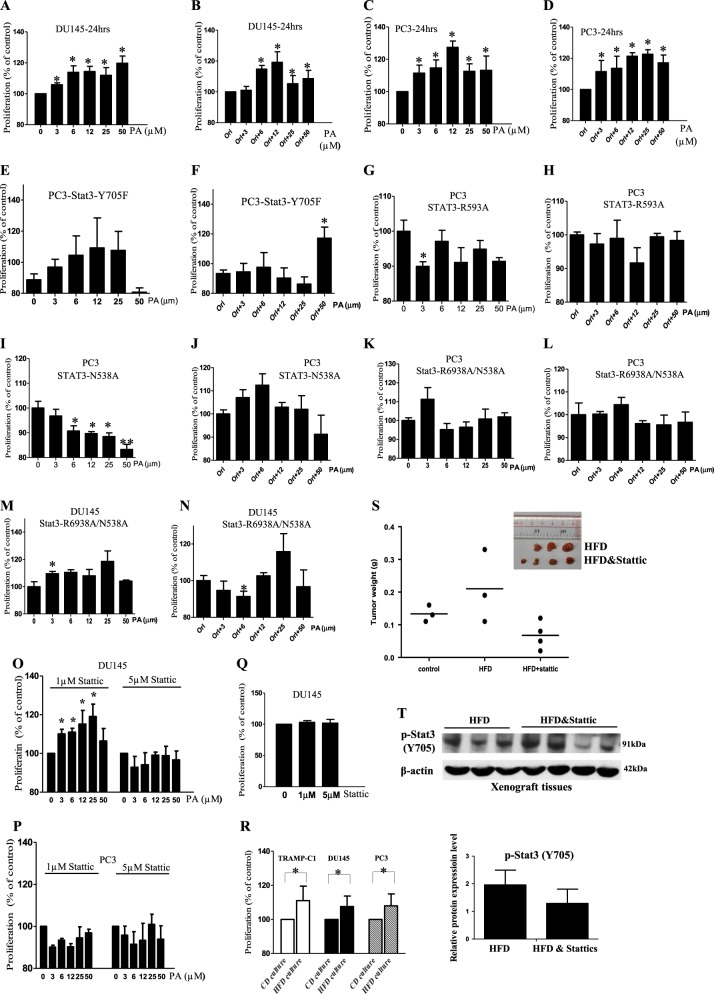
Palmitic acid (PA) increases prostate cancer (PCa) cell proliferation which is mediated by signal transducer and activator of transcription-3 (STAT3) activity. Proliferation of **a** DU145, **c** PC3 cells upon PA challenge at the indicated concentrations for 24 h. Proliferation of **b** DU145 and **d** PC3 cells after PA treatment for 24 h in the presence of orlistat (Orl, 1 µM). Proliferation of PC3 cells overexpressed with Stat3-Y705F treated by PA at the indicated concentrations for 24 h **e** in the absence or **f** presence of Orl (1 µM). Proliferation of PC3 cells overexpressed with Stat3-R593A treated by PA at the indicated concentrations for 24 h **g** in the absence or **h** presence of Orl (1 µM). Proliferation of PC3 cells overexpressed with STAT3-N538A treated by PA at the indicated concentrations for 24 h **i** in the absence or **j** presence of Orl (1 µM). Proliferation of PC3 cells overexpressed with Stat3-R6938A/N538A treated by PA at the indicated concentrations for 24 h **k** in the absence or **l** presence of Orl (1 µM). Proliferation of DU145 cells overexpressed with Stat3-R6938A/N538A treated by PA at the indicated concentrations for 24 h **m** in the absence or **n** presence of Orl (1 µM). Proliferation of **o** DU145 and **p** PC3 cells under PA challenge in the presence of stattic. **q** Proliferation of DU145 cells in the presence of stattic at the indicated concentrations. **r** Proliferation of DU145, PC3, and TRAMP-C1 cells after culturing with the serum of the HFD-feeding or control diet (CD)-feeding mice. CD culture, cells cultured with serum of CD-feeding mice; HFD culture, cells cultured with serum of HFD-feeding mice. Shown is the mean ± SEM, *n* = 3 independent experiments. *p value <0.05. **s** Tumor weight of the DU145-bearing xenograft mouse model fed by match CD or HFD with or without stattic treatment (2.5 mg/kg). **t** Western blots of phosphorylated (p)-STAT3 (Y705) in the xenograft tissues of the HFD-feeding xenograft mouse model with or without stattic treatments (2.5 mg/kg). Shown is the mean ± SEM, *n* = 3–4 mice in each group

Next, we investigated whether PA affected PCa cell proliferation, and whether STAT3 activity was involved. We found that PA ranged from 3 to 50 µM significantly increased PCa cell proliferation after 24-h treatment (Fig. 5a, c) and after 48-h treatment (Supplementary Fig. S2A, C). To discriminate the proliferative effect of endogenous and exogenous PA, we pre-treated the PCa cells with orlistat, which inhibits a critical lipogenic enzyme fatty acid synthase. We found that in the presence of orlistat, exogenous PA could also significantly increase the PCa cell proliferation (Fig. 5b, d and Supplementary Fig. S2B, D), suggesting that exogenous PA increases PCa cell proliferation. A similar result was obtained with TRAMP-C1 cells (Supplementary Fig. S2E–H). PA treatment did not induce apoptosis in the PCa cells (Supplementary Fig. S3A, B).

Next, we performed in vitro studies to suggest the involvement of STAT3 in mediating the PA-enhanced PCa cell proliferation. We overexpressed PCa cells with the dominant-negative STAT3-Y705F or the STAT3 mutants. Data showed that PA did not significantly increase the proliferation of the PCa cells that were overexpressed with STAT3-Y705F (Fig. 5e, f) or those overexpressed with STAT3-R593A (Fig. 5g, h) or STAT3-N538A (Fig. 5i, j) or double mutations R593A + N538A (Fig. 5k, n). Besides, we also pre-treated the PCa cells with STAT3 inhibitor STA before PA treatment. Inhibition of STAT3 activity significantly reduced the PA-increased cell proliferation in both DU145 cells (Fig. 5o) and PC3 cells (Fig. 5p). STA had no significant effect on the proliferation of the control PCa cells (Fig. 5q). STA did not induce apoptosis in the PCa cells as revealed by the apoptotic marker, cleaved poly(ADP-ribose) polymerase (PARP) (Supplementary Fig. S4). In the ex vivo setting, we found that PCa cell proliferation was significantly increased after the cells were cultured with HFD-feeding mouse serum (Fig. 5r). We also use xenograft mouse model to further suggest the involvement of STAT3 in the HFD-increased PCa growth. We fed the mice HFD, and when the tumors were grown to the size of ~80 mm^3^, we used STAs to inhibit STAT3 in the tumors^22^. We found that inhibition of STAT3 in these tumors reduced tumor growth (Fig. 5s) and STAT3 phosphorylation (Fig. 5t). Besides, we also used formulated diet that was rich in PA (PAD) (Research Diets) to feed the DU145-bearing xenograft mouse model. Data showed that PAD-feeding increased tumor growth compared to the matched CD-feeding, which was reversed by STAT3 inhibition (Supplementary Fig. S5).

Taken together, these data imply that STAT3 activity mediates the PA-increased PCa growth both in vitro and in vivo.

### PA increases PCa cell migration and invasion, which are mediated by STAT3 activity

**Fig. 6:**
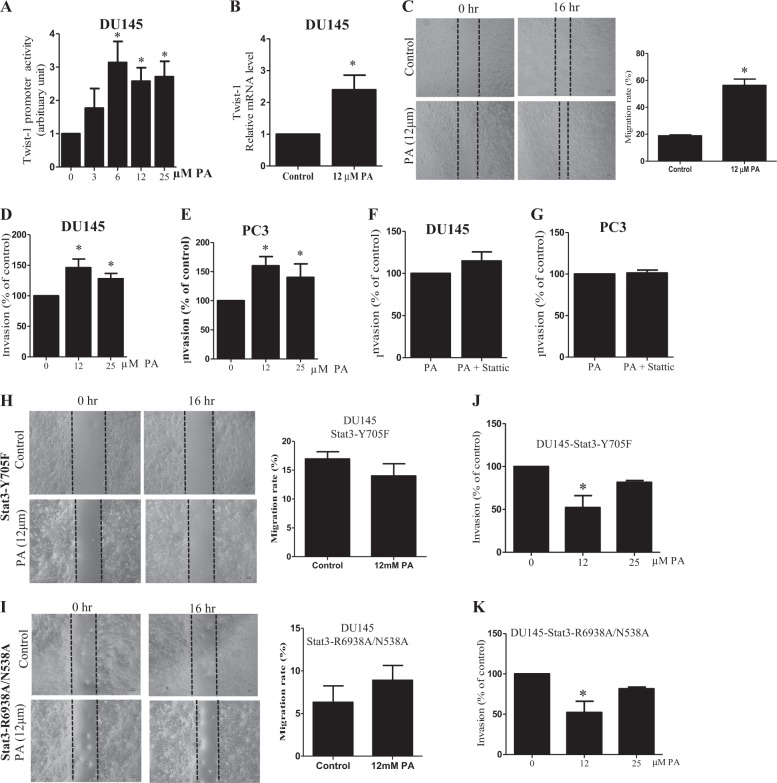
Palmitic acid (PA) increases prostate cancer (PCa) cell migration and invasion which are mediated by signal transducer and activator of transcription-3 (STAT3) activity. **a** Twist-1 luciferase reporter activity in DU145 cells upon PA challenge. **b** Relative messenger RNA (mRNA) level of Twist-1 in DU145 cells upon PA challenge. **c** Scratches in confluent monolayer of DU145 cells in the presence or absence of 12 µM PA at 0 and 16 h. Transwell invasion assay showing the invasive capacity of DU145 cells upon PA challenge **d** without or **f** with 5 µM stattic. Transwell invasive capacity of PC3 cells upon PA challenge **e** with or **g** without 5 µM stattic. Scratches in confluent monolayer of DU145 cells overexpressed with **h** Stat3-Y705F and **i** Stat3-R6938A/N538A in the presence or absence of 12 µM PA at 0 and 16 h. Transwell invasion assay showing the invasive capacity of DU145 cells overexpressed with **j** Stat3-Y705F and **k** Stat3-R6938A/N538A upon PA challenge. Shown is the mean ± SEM, *n* = 3 independent experiments. *P value < 0.05

We also found that PA increased the promoter activity of Twist-1, a STAT3 target gene (Fig. 6a). The treatment increased Twist-1 mRNA level (Fig. 6b) in DU145 cells. Since twist-1 correlates with invasive and metastatic lesions in PCa^23^, we examined whether migration and invasion were affected by PA treatments. As shown in Fig. 6c, PA significantly increased DU145 cell migration. PA also increased invasion of DU145 cells (Fig. 6d) and PC3 cells (Fig. 6e), which was abolished in the presence of STA (Fig. 6f, g). To further suggest the involvement of STAT3 in mediating the PA-enhanced PCa cell migration and invasion, we overexpressed PCa cells with dominant-negative STAT3-Y705F or the STAT3 mutant R593A + N538A. Data showed that PA did not significantly increase the migration (Fig. 6h) or invasion (Fig. 6j) of the PCa cells that were overexpressed with STAT3-Y705F or those overexpressed with R593A + N538A (Fig. 6i, k). These data suggest that PA increases PCa cell migration and invasion in PCa cells, which is mediated by STAT3 activity.

## Discussion

The progression from non-castration-resistant PCa (non-CRPC) to CRPC usually occurs within 2–3 years^24^. In this study, we used both human androgen-sensitive and castration-resistant PCa cells to investigate whether STAT3 activity is involved in the HFD-enhanced PCa growth. Our data showed that HFD feeding increased tumor size, STAT3 phosphorylation (Y705), and PA levels in the xenograft tissues of the PCa-bearing mouse model. Subsequent in vitro studies showed that PA enhanced STAT3 phosphorylation at Y705, and to a less extent at Y727. The increase in STAT3 phosphorylation may be underlined by two mechanisms of action. First, PA increased STAT3 mRNA and protein levels, which then increased the amount of the p-STAT3-Y705. Second, computational modeling suggests strong and stable binding between PA and USTAT3 at R593 and N538, which changes the STAT3 structure and affects its activity. Indeed, functional studies showed that STAT3 mutants R583A and N538A significantly reduced PA-enhanced STAT3 phosphorylation, and the PA-increased PCa cell proliferation, migration, and invasion. The role of STAT3 in PA-enhanced PCa growth is also suggested in animal studies in which HFD or PAD increased PCa growth and was reversed by STAT3 inhibitor.

Here, we have identified p-STAT3-Y705, which drives the HFD-associated PCa growth even the PCa may have different endogenous STAT3 levels. PC3 cells have been reported to have no endogenous STAT3^25,26^ or express endogenous STAT3^8,27^. Indeed, our data showed that PC3 has a relatively low basal level of STAT3 compared to other PCa cell lines. Nevertheless, upon PA challenge, p-STAT3-Y705 is significantly increased in the PC3 cells. Similarly, in Mora et al.^8^ study, the very low expression of STAT3 in PC3 and LNca cells can be significantly increased upon IL-6 stimulation. Our data suggest that the role of STAT3 in mediating HFD-associated PCa growth is not affected by the basal level of STAT3 in the PCa.

In other cancer types^28–32^, activation of STAT3 by phosphorylating at Y705 is known to be mediated by EGFR, JAK2, or ERK activities, or by IL-6 or leptin stimulation. Interestingly, our data suggest that PA may directly regulate STAT3 activity. Indeed, a previous study showed that the signal-transducing adaptor protein-2 (STAP-2), which contains a YXXQ motif in the C-terminal region, serves as a potent STAT3-binding site and that STAP-2 binds to STAT3 and activates STAT3^33^. Our data suggest that there are strong and stable bindings between PA and the USTAT3, which lead to a change of the relative positions of subunits A and B. PA induces rotation of the two subunits and tend to have the same horizontal plane, while the STA, a STAT3 inhibitor, stabilizes the relative positions of subunit A and B. Thus, we speculate that the changes of USTAT3 structure is involved in the activation of STAT3 upon PA challenge. Our speculation is further supported by our finding that the computational homology model of human STAT3 dimer has the subunits A and B of STAT3 kept in the same horizontal plane, which resembles the activated STAT3. Furthermore, we found that the TYR-640, GLU-637, and SER-636 in SH2 domain and the THR-708, LEU-706, and TYR-705 in transactivaion domain are involved in the interaction of the two subunits. Of note, TYR-705 has been demonstrated as the key residue for STAT3 dimer formation. These results strongly suggest that the structural change of USTAT3 induced by PA underlies the activation of STAT3 upon the PA challenge. Furthermore, STAT3 mutants with the potential PA-binding sites removed could not be phosphorylated by PA, and the PA-enhanced cell proliferation, migration, and invasion are reduced. These results strongly suggest that these potential binding sites on STAT3 are important in mediating the PA effects.

Unlike many other cancer cells, PCa cells mainly rely on the uptake and metabolism of fatty acids^7^. Our study also suggests that the uptake of fatty acids varies between different PCa cell lines; DU145 cells uptake both PA and stearic acids from the HFD-fed mouse serum, while PC3 and TRAMP-C1 cells do not uptake stearic acid but only PA. A possible reason is that DU145 cells takes up fatty acid-binding protein 4 that increases fatty acid binding^34^, which may facilitate the uptake of 18C stearic acid.

In PCa, both clinical and experimental studies have revealed the elevated expressions of STAT3 and its target genes in clinical samples, which are positively correlated with the aggressiveness and frequency of the metastasis in patients^10,11^. Our data suggest that a regulatory role of the PA/STAT3 axis in HFD-associated PCa growth, which will have great implication because PA is the dominant saturated fatty acid in HFD. Our data not only provide insight into the pathogenesis of HFD-associated PCa but also open new avenues for the study of other HFD-associated cancer growth in relation to STAT3 activity.

## Methods

### Establish DU145-bearing xenograft mouse model with dietary intervention

All animal experimentation was approved and conducted in accordance with the guidelines from Hong Kong Baptist University and was endorsed by the University Human and Animal Subject Committee and the Department of Health, the Government of Hong Kong Special Administration Region. Male nude mice of 4 weeks old were purchased from the Chinese University of Hong Kong. DU145 cells (ATCC) were washed in phosphate-buffered saline (PBS) containing 5 mM EDTA, the cell number and viability were examined using trypan blue. Single-cell suspension of >90% viability was suspended at 1 × 10^5^ cells in 100 µl PBS and inoculated subcutaneously into each mouse. When the tumor grows to ~80 mm^3^ in size, mice were randomly selected to have either HFD (D12492, 60 kcal% fat, Research Diets) or the corresponding matched CD (D12450J, 10 kcal% fat 7% sucrose, a matched control for D12492, Research Diets). Alternatively, they had a diet rich in PA (D16042106) or the corresponding matched CD (D17041705). All these diets are formulated by Research Diets, Inc. The fatty acid compositions of these diets are shown in Supplementary Table S1. STA treatment^22^ was given at e dose of 2.5 mg/kg when the dietary intervention started. Both diet and water were supplied ad libitum. Body weight and tumor size of the xenograft mouse models were measured every day.

### LC/MS-based lipidomics

For the sample preparation, blood samples were withdrawn by heart puncture and xenografts were dissected after mice were scarified. For lipid extraction, to each 0.2 ml serum sample were added 0.3 ml of 0.5 M KH_2_PO_4_, 1.5 ml of chloroform, and 0.5 ml of methanol. After vortex for 2 min and centrifugation at 2000 × *g*, the lower phase was collected and evaporated under a nitrogen stream. The residue was reconstituted in 10 μl of isopropanol, diluted with 90 μl of methanol, and subjected to free fatty acid and phospholipid analyses^35,36^. Xenograft samples (100 mg) were homogenized using Polyton PT 6600 in 750 μl of prechilled 10% trichloroacetic acid supplemented with 500 ng/ml of mixed internal standard hexadecanoic-15,15,16,16,16-*d*_5_ acid (CDN Isotopes, Canada). The tissue homogenates were centrifuged at 14,500 × *g* for 10 min at 4 °C. The resulting supernatants were then extracted twice with 5 ml of chloroform/methanol solution (v/v = 2:1, Folch reagent) as described^35,36^. An Agilent 6540 UHD Accurate-Mass Q-TOF LC/MS (quadrupole time-of-flight liquid chromatography-mass spectrometry) mass spectrometer (Agilent Technologies) was connected to an Agilent 1290 Infinity UHPLC via an ESI (electrospray ionization) ion source for the analysis of total lipids. An Agilent 6450 Triple Quadrupole LC/MS system accompanied with MassHunter Workstation software (version B.04.00 Qualitative Analysis, Agilent Technologies, Santa Clara, CA, USA) was connected to an Agilent 1290 Infinity UHPLC for specific quantification of the lipids^35,36^.

### LC/MS data processing and statistical analysis

With the MassHunter Workstation software (version B.04.00 Qualitative Analysis, Agilent Technologies, Santa Clara, CA), ions were extracted by molecular features characterized by retention time (RT), intensity in apex of chromatographic peak, and accurate mass. These results were then analyzed by Mass Profiler Professional (MPP) software (version 2.2, Agilent Technologies, Santa Clara, CA). We first set up a filtration platform to further filter the initial entities before doing PCA. Only entities with abundance above 3000 cps were selected. These entities were then passed a tolerance window of 0.15 min and 2 mDa chosen for alignment of RT and *m*/*z* values, respectively. The data were also normalized by internal standard. We employed one-way analysis of variance (ANOVA) to do the statistical analysis. The *p* value of ANOVA was set to 0.05 (corresponding with the significance level of 95%). We also performed fold change (FC) analysis to further filter the entities, and only those entities with FC >2 were selected.

### Cell culture

Human PCa cells (DU145, PC3, and LNCaP cell lines) and murine PCa cells (TRAMP-C1 cell line) were purchased from the American Type Culture Collection (ATCC, USA) and maintained in full medium, which was high glucose Dulbecco’s modified Eagle’s medium (Thermo Fisher Scientific, USA) supplemented with 10% fetal bovine serum (Thermo Fisher Scientific, USA) and 1% penicillin/streptomycin (Thermo Fisher Scientific, USA) in a humidified 5% CO_2_ atmosphere at 37 °C. In each experimental setup, equal number of cells were incubated for 24 h with equal volume of the mixed medium that contained 1:1 (v/v) in ratio of the full medium to mouse serum^16^. For PA treatment, cells were incubated with PA at the indicated concentration in the presence of 1% fatty acid-free bovine serum albumin (BSA) (Sigma-Aldrich), and 1% fatty acid-free BSA alone served as vehicle control. DU145 and PC3 cells were transfected with either Stat3-Y705F (Addgene), Stat3-R6938A, Stat3-N538A, or Stat3-R6938A/N538A plasmid using Lipofectamine 2000 (Invitrogen) according to the manufacturer’s instructions. Stat3-R6938A, Stat3-N538A, and Stat3-R6938A/N538A were constructed with QuikChange Site-Directed Mutagenesis Kit following the manufacturer’s instructions.

### Cell proliferation assay

We measured the cell proliferation with CyQUANT® Direct Cell Proliferation Assay Kit (Thermo Fisher Scientific, USA) following the manufacturer’s instruction. Briefly, PCa cells (15 × 10^4^ cells) were placed in each well of a 96-well plate. Proliferation assay was carried out 24 or 48 h after treatment. Fluorescent signals were detected by fluorescence excitation/emission maxima 480/535 nm.

### Quantification of apoptosis

Apoptotic cells were assessed by using Annexin V-Fluorescein Isothiocyanate Apoptosis Detection Kit I (BD Bioscience, USA) following the manufacturer’s instruction. Samples of 10,000 stained cells were analyzed by using a flow cytometer as previously described.

### Oil Red-O staining

Lipids are stained by Oil Red-O staining as previously described^37^. Briefly, to quantify staining, Oil Red-O was extracted from the cells with isopropanol containing 4% Nonidet P-40, and optical density was then measured at a wavelength of 520 nm.

### Real-time PCR

Total RNA was isolated using TRIzol reagent (Thermo Fisher Scientific, USA). Reverse transcription was performed with oligo-dT using MMLV reverse transcriptase (Promega, USA) according to the manufacturer’s protocol. Quantitative real-time PCR was carried out by monitoring the increase in fluorescence of SYBR green with the ViiA 7 Real-Time PCR System (Applied Biosystems, USA). The primer sets used were as follows: CD36, forward: AGATGCAGCCTCATTTCCAC, reverse: GCCTTGGATGGAAGAACAAA. TP53, forward: ACCTATGGAAACTACTTCCTGAAA, reverse: CTGGCATTCTGGGAGCTTCA. Cyclin D1, forward: AGCTGTGCATCTACACCGAC, reverse: GAAATCGTGCGGGGTCATTG. NF-κB, forward: GCAGATGGCCCATACCTTCA, reverse: CACCATGTCCTTGGGTCCAG. GATA2, forward: ATCAAGCCCAAGCGAAGACT, reverse: CATGGTCAGTGGCCTGTTAAC. β-Catenin, forward: CTGCTGTTTTGTTCCGAATGT, reverse: CCATTGGCTCTGTTCTGAAG. SERBP1, forward: CAAGGCCATCGACTACATT, reverse: TTGCTTTTGTGGACAGCAGT. PPAR-γ, forward: GCCCTTTGGTGACTTTATGGA, reverse: GCAGCAGGTTGTCTTGGATG. STAT3, forward: CCTGAAGCTGACCCAGGTAGC, reverse: CACCTTCACCATTATTTCCAAAC. NRF-1, forward: GCTGGACACCATCCTGAATC, reverse: CCTTCTGCTTCATCTGTCGC. N-myc, forward: TCCACCAGCAGCACAACTATG, reverse: GTCTAGCAAGTCCGAGCGTGT. c-Myc, forward: AAAGGCCCCCAAGGTAGTTA, reverse: GCACAAGAGTTCCGTAGCTG. β-Actin, forward: CATGTACGTTGCTATCCAGGC, reverse: CTCCTTAATGTCACGCACGAT. Each sample was amplified in triplicate for quantification. Data were analyzed by relative quantitation using the ∆∆Ct method, where ΔCt is the difference in threshold cycle values between the targets and β-actin, and ∆∆Ct is the difference between the treatment and vehicle control groups. All samples were analyzed in triplicate and normalized to actin.

### Luciferase assay

Luciferase assay was done as described previously^38^. Briefly, PCa cells were seeded in 24-well plates and transiently co-transfected with STAT3 reporter plasmid 4xM67 pTATA TK-Luc or Twist-1 promoter reporter construct Twist-1-Luc (0.2 mg/per well, Addgene, USA) with Renilla luciferase-expressing plasmid PRLCMV (0.1 mg/per well, Promega, USA) by use of Lipofectamine 2000 (Thermo Fisher Scientific, USA). After 48 h, cells were treated with PA at the indicated concentration with 1% fatty acid-free BSA for 24 h, and then lysed in 100 ml of passive lysis buffer (Promega, USA) in each well. A 25-ml aliquot of cell lysate was subjected to a luciferase assay by using the Dual Luciferase Assay Kit (Promega, USA). STAT3-luciferase activity was measured by EnVision Mutilabel Reader (Perkin-Elmer, USA). Relative luciferase activity was calculated after the activity of STAT3-dependent firefly luciferase had been normalized to that of Renilla luciferase. All values are expressed as fold induction relative to basal activity.

### Western blotting analysis

The nitrocellulose membrane (Amersham, Arlington Heights, IL, USA) carrying transferred proteins was incubated at 4 °C overnight with corresponding antibody at 1:1000 ratio. Antibodies against STAT3, phospho-STAT3 (TYR-705), phospho-STAT3 (Y727), JAK2, phospho-JAK2 (Y1007/1008), JAK2, phospho-EGFR (Y845), phospho-EGFR (Y992), phospho-EGFR (Y1068), phospho-EGFR (Y1148), phospho-ERK (42/44 kDa), IL-6, phosphorylated leptin receptor, β-actin, and glyceraldehyde 3-phosphate dehydrogenase (GAPDH) were obtained from Cell Signaling Biotechnology or Abcam, USA. Immunodetection was accomplished using horseradish peroxidase-conjugated secondary antibody, followed by detection by ECL (Amersham, USA).

### Molecular simulation

Molecular docking was performed to clarify the binding mechanism between USTAT3 (PDB ID: 3CWG) with PA (ZINC ID: 6072466) or STA (ZINC ID: 162014). The protein for molecular docking simulation was prepared by removing water molecules and bound ligands. The binding pocket between USTAT3 subunits was defined in this study as the protein–ligand binding site of STAT3. ICM-Pro was utilized for molecular docking. PyMol was used to visualize the USTAT3-PA and USTAT3-STA complexes. MD simulation was performed by YASARA, and the AMBER 03 forcefield was used to run all simulations. Specifically, 0.9% NaCl served as solvation of the receptor–ligand complex in a dodecahedron box, with a distance of 5 Å between the box and the solute. The initiation of simulated annealing minimizations was set at 298 K, with velocities scaling down with 0.9 every ten steps lasting for 5 ps. Following energy minimization, temperature of the system was adjusted utilizing a Berendsen thermostat to minimize the influence of temperature control. In addition, velocities were rescaled every 100 simulation steps, whenever the mean of last 100 detected temperatures converged. Finally, 50 ns MD simulations were conducted at a rate of 2 fs, and the coordinates of the complexes were saved every 10 ps.

The SWISS-MODEL, a homology-modeling tool, was used to generate a homology model of human STAT3. The human amino acid sequence of STAT3 (NP_644805.1) was obtained from NCBI. The three-dimensional crystal structure of the mouse STAT3 monomer (PDB ID: 1BG1) was employed as template for homology modeling. MD simulation was performed according to above methods to verify the stability of the STAT3 homology model.

### Wound healing assay

Wounds were created by scratching the confluent cell monolayer using a plastic pipette tip, and any loose cellular debris or detached cells were removed by PBS wash. The cells were pre-treated with mitomycin at 5 µg/ml for 24 h. Then, these cells were treated with PA at 12.5 µM with 1% fatty acid-free BSA or 1% fatty acid-free BSA alone as vehicle control for 16 h. The gaps of the wounds were observed with phase contrast microscopy and digitally photographed under 100 × magnifications.

### Transwell invasion assay

Cell invasion was determined by using BD BioCoat™ Matrigel™ invasion chamber (BD Biosciences) according to the manufacturer’s instruction. In brief, 0.75 ml of serum-starved culture medium was added to the lower chamber along with chemoattractants, and aliquots of 0.5 ml of cell suspensions consisting of 5 × 10^5^ cells/ml in Dulbecco's modified Eagle’s medium containing PA at 12.5 or 25 µM with 1% fatty acid-free BSA or 1% fatty acid-free BSA were seeded on upper wells and allowed to invade for 24 h. The non-invading cells were removed by scrubbing with cotton swab and the cells on the lower surface of the membrane were stained with crystal violet. The invasive capacity was quantified by counting the cells that migrated to the lower side of the filters in different fields under a microscope at ×200. Data were expressed as the percentage of invasion in control.

### Immunohistochemical staining

The xenograft tissues were fixed in 4% paraformaldehyde, embedded in paraffin, and then sliced up (4 μm). The slices were stained with the corresponding antibody, or with hematoxylin and eosin before being examined and quantified.

### Statistical analysis

All data are expressed as mean ± standard error of the mean. Statistical analysis was performed by the Student’s *t* test, using the statistical software GraphPad Prism 5.0. *P* < 0.05 was considered statistically significant. *P value < 0.05; **p value < 0.01.

## Supplementary information

Supplementary figure legends

Supplementary Figures

Supplementary Tables
