# Supplementary material for: Signal transducer and activator of transcription-3 drives the high-fat diet-associated prostate cancer growth
Source: Cell Death Dis. 2019 Sep 2;10(9):637. doi: 10.1038/s41419-019-1842-4 (PMC6717738; doi:10.1038/s41419-019-1842-4)
Supplement: Supplementary file 1 — Supplementary figure legends [file 41419_2019_1842_MOESM1_ESM.doc]

**Supplementary Figure S1**

Representative western blots showing the levels of **(A)** JAK2, JAK2 phosphorylation (pJAK2-Y1007/1008), **(B)** phosphorylation of EGFR (pEGFR-Y845, Y992, Y1068 and Y1148), **(C)** phosphorylation of ERK (42/44kDa), **(D)** IL6 and **(E)** p-ObR in PC3 and DU145 cells upon PA challenge for 24 hr. JAK, Janus kinase; EGFR, epidermal growth factor receptor; ERK, extracellular signal-regulated kinase; IL6, interleukin 6; p-ObR, phosphorylated leptin receptor.

**Supplementary Figure S2**

Proliferation of **(A)** DU145, **(C)** PC3 cells upon PA challenge at the indicated concentrations for 48 hr. Proliferation of **(B)** DU145, **(D)** PC3 cells after PA treatment for 48 hr in the presence of orlistat (Orl, 1µM). Proliferation of TRAMP-C cells upon PA challenge at the indicated concentrations for **(E)** 24 hr and **(G)** 48 hr. Proliferation of TRAMP-C cell the after PA treatment for **(F)** 24 hr and **(H)** 48 hr in the presence of orlistat (Orl, 1µM).

**Supplementary Figure S3**

PA at 25-50 µM range does not induce apoptosis in PCa cells. **(A)** Annexin V-fluroescein isothiocyanate (FITC)/propidium iodine (PI) staining of DU145 cells upon PA challenge. **(B)** Summary of 3 individual sets of data showing the percentage of total apoptotic cells. Shown is the mean ± SEM, n = 3 independent experiments.

**Supplementary Figure S4**

Representative western blot of cleaved PARP in PC3 cells after treating with stattic at the indicated concentrations for 24 hr. PARP, poly (ADP-ribose) polymerase.

**Supplementary Figure S5**

Tumor volume of the xenograft mouse model fed with matched control diet (CD), or fed with palmitic acid-rich diet (PAD) with or without stattic (2.5mg/kg) treatment. Shown is the mean ± SEM, 4 mice in each group. **p* value <0.05.
