# Supplementary material for: Signal transducer and activator of transcription-3 drives the high-fat diet-associated prostate cancer growth
Source: Cell Death Dis. 2019 Sep 2;10(9):637. doi: 10.1038/s41419-019-1842-4 (PMC6717738; doi:10.1038/s41419-019-1842-4)
Supplement: Supplementary file 2 — Supplementary Figures [file 41419_2019_1842_MOESM2_ESM.docx]

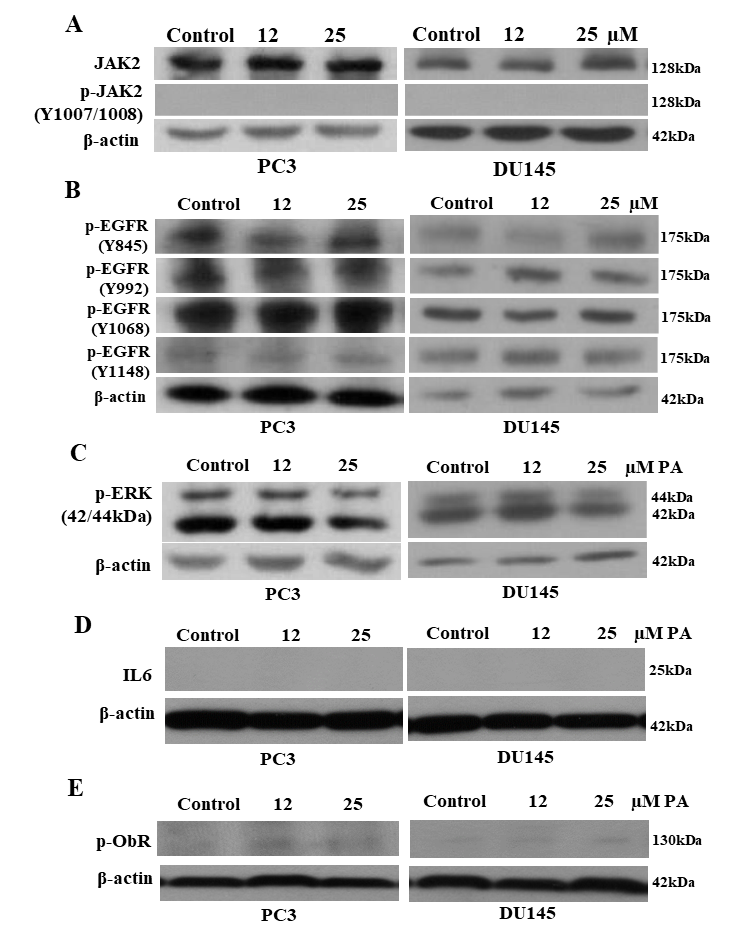


**Supplementary Figure S1**


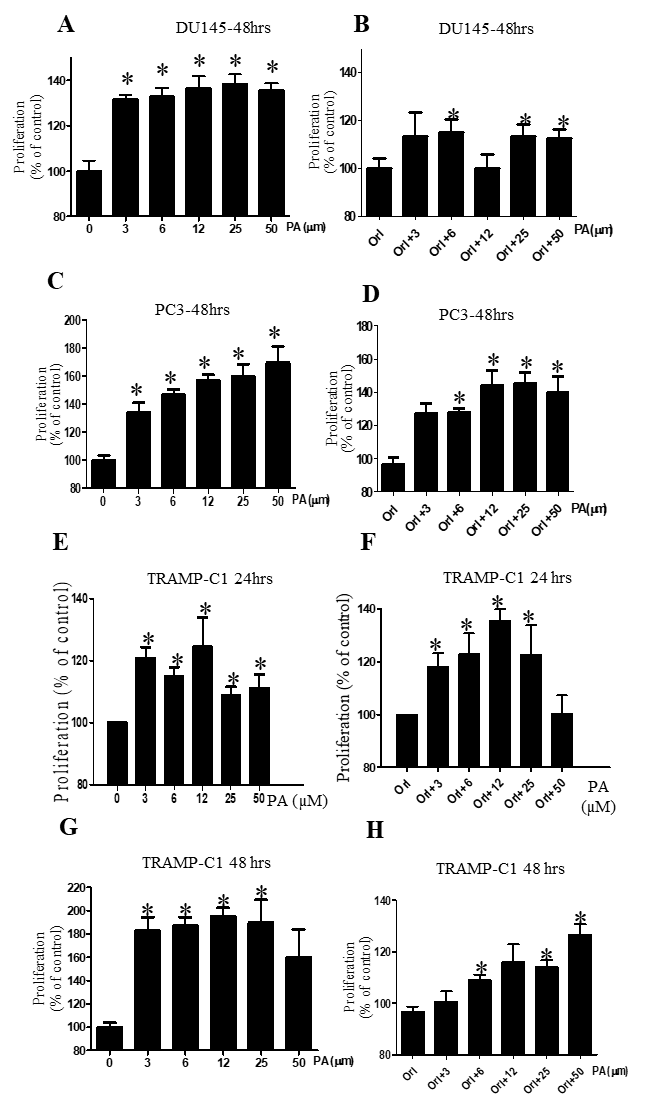


**Supplementary Figure S2**


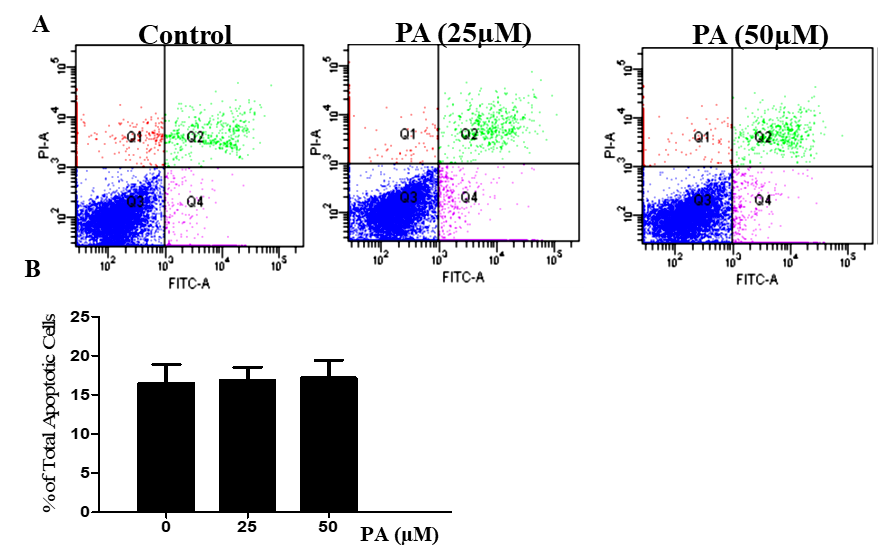


**Supplementary Figure S3**


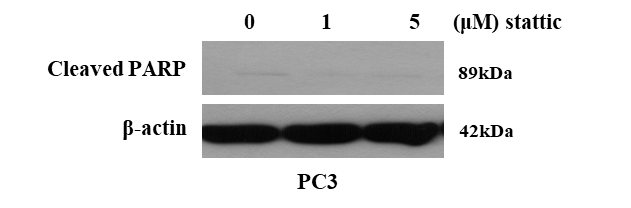


**Supplementary Figure S4**


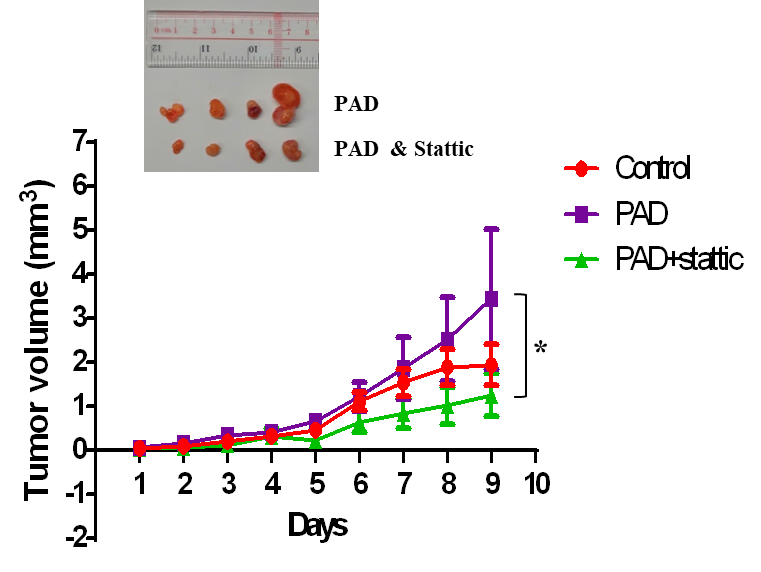


**Supplementary Figure S5**
