# Supplementary material for: Signal transducer and activator of transcription-3 drives the high-fat diet-associated prostate cancer growth
Source: Cell Death Dis. 2019 Sep 2;10(9):637. doi: 10.1038/s41419-019-1842-4 (PMC6717738; doi:10.1038/s41419-019-1842-4)
Supplement: Supplementary file 3 — Supplementary Tables [file 41419_2019_1842_MOESM3_ESM.doc]

**Supplementary Table S1**

**Fatty acid profiles of the diets**

**Supplementary Table S2**

**Changes in the lipid species in the xenograft tissues of the high fat diet (HFD)-feeding mice**

**Supplementary Table S2 (cont’d)**

**Changes in the lipid species in the xenograft tissues of the high fat diet (HFD)-feeding mice**
